# Identification of differences in CD4^+^ T-cell gene expression between people with asthma and healthy controls

**DOI:** 10.1038/s41598-023-49135-9

**Published:** 2023-12-20

**Authors:** Mauro Tutino, Jenny Hankinson, Clare Murray, Lesley Lowe, Gina Kerry, Magnus Rattray, Adnan Custovic, Sebastian L. Johnston, Chenfu Shi, Gisela Orozco, Stephen Eyre, Paul Martin, Angela Simpson, John A. Curtin

**Affiliations:** 1 Division of Infection, Immunity and Respiratory Medicine, School of Biological Sciences, Faculty of Biology, Medicine and Health, University of Manchester, Manchester, M13 9PL UK; 2 Manchester Biomedical Research Centre, Manchester University NHS Foundation Trust, Manchester, UK; 3 Division of Informatics, Imaging and Data Sciences, School of Biological Sciences, Faculty of Biology, Medicine and Health, University of Manchester, Manchester, UK; 4 National Heart and Lung Institute, Asthma UK Centre in Allergic Mechanisms of Asthma, Imperial College London, London, UK; 5 Centre for Genetics and Genomics Versus Arthritis, Division of Musculoskeletal and Dermatological Sciences, School of Biological Sciences, Faculty of Biology, Medicine and Health, The University of Manchester, Manchester, UK; 6 The Lydia Becker Institute of Immunology and Inflammation, Faculty of Biology, Medicine and Health, University of Manchester, Manchester, UK

**Keywords:** RNA sequencing, Asthma, Gene expression, Gene regulatory networks

## Abstract

Functional enrichment analysis of genome-wide association study (GWAS)-summary statistics has suggested that CD4^+^ T-cells play an important role in asthma pathogenesis. Despite this, CD4^+^ T-cells are under-represented in asthma transcriptome studies. To fill the gap, 3'-RNA-Seq was used to generate gene expression data on CD4^+^ T-cells (isolated within 2 h from collection) from peripheral blood from participants with well-controlled asthma (n = 32) and healthy controls (n = 11). Weighted Gene Co-expression Network Analysis (WGCNA) was used to identify sets of co-expressed genes (modules) associated with the asthma phenotype. We identified three modules associated with asthma, which are strongly enriched for GWAS-identified asthma genes, antigen processing/presentation and immune response to viral infections. Through integration of publicly available eQTL and GWAS summary statistics (colocalisation), and protein–protein interaction (PPI) data, we identified *PTPRC*, a potential druggable target, as a putative master regulator of the asthma gene-expression profiles. Using a co-expression network approach, with integration of external genetic and PPI data, we showed that CD4^+^ T-cells from peripheral blood from asthmatics have different expression profiles, albeit small in magnitude, compared to healthy controls, for sets of genes involved in immune response to viral infections (upregulated) and antigen processing/presentation (downregulated).

## Introduction

Asthma is a chronic inflammatory condition of the airways. Childhood asthma, usually linked with allergy and IgE production, has historically been associated with a Th2/Th1 imbalance with increased Th2 CD4^+^ T-cell mediated responses and Th2 cytokines such as IL-4, IL-5 and IL-13^1^. In recent years the so called Th2 hypothesis has been questioned by the observation of increased cell proportions of other CD4^+^ cell types in the lungs of severe asthmatics, such as Th1^2,3^ and Th17^3^. Regulatory T-cells (Treg), a type of CD4^+^ T-cell important in the negative regulation of inflammatory responses, were also found to be decreased in bronchoalveolar lavage fluid (BALF) from asthmatic children compared to healthy controls^4^.

Further evidence of the importance of CD4^+^ T-cells in asthma comes from large GWAS studies, which showed high enrichment for CD4^+^ T-cell specific enhancer marks^5^, regions of open chromatin^6^ and gene sets^7^.

It is therefore widely recognised that CD4^+^ T-cells play an important role in asthma pathogenesis. Despite this, only one study specifically targeted CD4^+^ T-cells^8^. The authors measured CD4^+^ gene expression using Affymetrix microarrays in 12 severe asthmatics and 8 healthy controls from an adult population (average age 46 years) and identified a small number (n = 40) of differentially expressed genes. It is currently not known if these differences can also be identified in patients with controlled asthma or if these are specific for a severe phenotype.

For the current study, we sought to employ RNA-sequencing, a more sensitive technique compared to microarray, in a larger sample size to study the transcriptome of CD4^+^ T-cells from well-characterised childhood asthma patients from the Manchester Asthma and Allergy Study cohort (MAAS) cohort^9^ with well-controlled asthma and healthy controls.

## Results

### Study population

The characteristics of the study population are shown in Table 1. As expected, asthmatics showed higher median values for measures of inflammation (fractional exhaled nitric oxide [FeNO]) and lower lung function measurements (baseline forced exhalation volume in 1 s/forced vital capacity [FEV_1_/FVC]) compared to controls. Moreover, 59% of participants with asthma were positive for house dust mite skin prick test, compared to only 25% of healthy controls.

**Table 1:** Table of subject characteristics.

	N	Asthmatics** N = 33	Controls N = 12	p-value
Female (%)	45	11 (33)	6 (50)	0.3
Current smoker (%)	45	3 (9.1)	0 (0)	0.6
Median FeNO (ppb, IQR)	45	24 (18–38)	14 (12–18)	0.008
Positive mannitol challenge (%)	42	17 (57)	3 (25)	0.063
Median PD_15_ (IQR)*	20	187 (51–258)	554 (383, 584)	0.093
Median FEV_1_/FVC (IQR)	45	84.0 (80.0–87.8)	87.3 (84.1–89.7)	0.088
Median BMI (IQR)	45	23.9 (21.6–25.8)	21.7 (20.3–23.2)	0.1
HDM SPT age 18	44	19 (59%)	3 (25%)	0.04
Age (years)	45	22.4 (21.8–22.7)	23.0 (22.8–23.1)	0.002
Medication				
None (%)	33	9 (27)		
Inhaled corticosteroids (ICS) (%)		9 (27)		
Combination Inhalers (contains both LABA and ICS) (%)		5 (15)		
SABA only (%)		10 (30)		

*IQR* 25th–75th interquartile range, *FeNO* fractional exhaled nitric oxide, *ppb* parts per billion, *FEV1* forced exhalation volume in 1 s, *FVC* forced vital capacity, *BMI* body mass index, *HDM* house dust mite, *SPT* skin prick test, *PD*_*15*_ dose of mannitol causing a 15% fall in FEV_1_, *LABA* long-acting bronchodilator inhalers, *SABA* short-acting bronchodilator inhalers.

*PD_15_ was only calculated for the participants who were positive to the mannitol challenge.

**Mannitol challenge was missing for 2 asthma cases; p value from Fisher’s exact test for categorical and Wilcoxon rank sum test for numerical variables.

### Weighted gene co-expression network analysis (WGCNA)

Two samples did not pass the initial QC and were excluded from the analysis (Supplementary Materials Fig. E1), leaving 32 asthmatics and 11 controls. Differential expression analysis with DESeq2 did not identify any differentially expressed genes (DEGs) (Supplementary Materials Fig. E2 and Supplementary Data S1). Post-hoc power calculation showed that, given the observed biological variation and read counts, a larger sample size (at least n = 52) would have been needed to achieve 80% power to detect differentially expressed genes with a fold change greater than 1.5 (see Supplementary Materials S1). Given the lack of power to detect DEGs, WGCNA was used instead. The network was built using the top 75% most variable genes (n = 9607) and identified 18 modules (Supplementary Materials Table E1, Supplementary Materials Figs. E3–E5 and ModuleGenes.xlsx S1). Logistic regression was used to identify associations between the module expression profiles and asthma status. Three modules were found to be associated with asthma (Table 2), the Green and Lightgreen modules, positively associated with asthma (i.e. the genes in these modules were generally over-expressed in asthmatics) and the Darkturquoise module, negatively associated with asthma. Sensitivity analyses adjusting for house dust mite skin prick test and exclusion of the three current smokers from the asthma group did not alter the results, suggesting that the identified module associations are not driven by atopy or smoking (see Supplementary Materials Table E3).

**Table 2:** WGCNA modules significantly associated with asthma.

	Beta	p-value	FDR
Green	6.81	0.02	0.14
Light green	7.05	0.02	0.14
Dark turquoise	− 12.40	0.01	0.14

### Over-representation analysis of asthma-associated module genes

The asthma-associated modules’ genes were found to be enriched in asthma related GWAS Catalog traits genes (Fisher’s exact test of enrichment for genes annotated to GWAS trait hits; Table 3). The Green module was enriched for genes associated with Childhood onset asthma/allergic disease (FDR = 0.01). The Darkturquoise module was also strongly enriched for asthma-associated genes (FDR = 4.4e−06), while the Lightgreen module was enriched for white blood cell (FDR = 0.03) and eosinophil counts (FDR = 0.01).

**Table 3:** Asthma modules’ genes enrichment in GWAS catalog traits at FDR < 0.05.

	GWAS trait	N	n	P-value	FDR
Green	Asthma (childhood onset)	266	14	6.8E−06	0.0118
	Allergic disease (asthma, hay fever or eczema)	292	14	1.9E−05	0.0118
Lightgreen	Eosinophil counts	186	7	9.4E−05	0.0114
	White blood cell count	221	7	2.7E−4	0.0309
	Sum eosinophil basophil counts	160	6	3.0E−4	0.0327
Darkturquoise	Asthma	337	11	2.9E−08	4.4e−06

N = number of genes in the gene set; n = number of module genes overlapping the gene set.

Query of the top 10 asthma-associated genes (by gene-significance [GS], see “Methods” for description) in each module (Supplementary Materials Fig. E6) in the GWAS Catalog and UK Biobank databases showed that a large proportion of the genes were associated with asthma, allergy and infection of the airways (Supplementary Materials Table E2). For the Green module, 7 out of 10 genes have been previously associated with asthma/allergy or viral infections. The top 10 Darkturquoise module genes were associated with asthma, prescription of allergic medication and infections of the airways. The Lightgreen module genes were mostly non-coding genes and most of them were not reported in the databases utilised. Those that were reported in the databases were associated with lung function and eosinophil counts.

**Figure 1:**
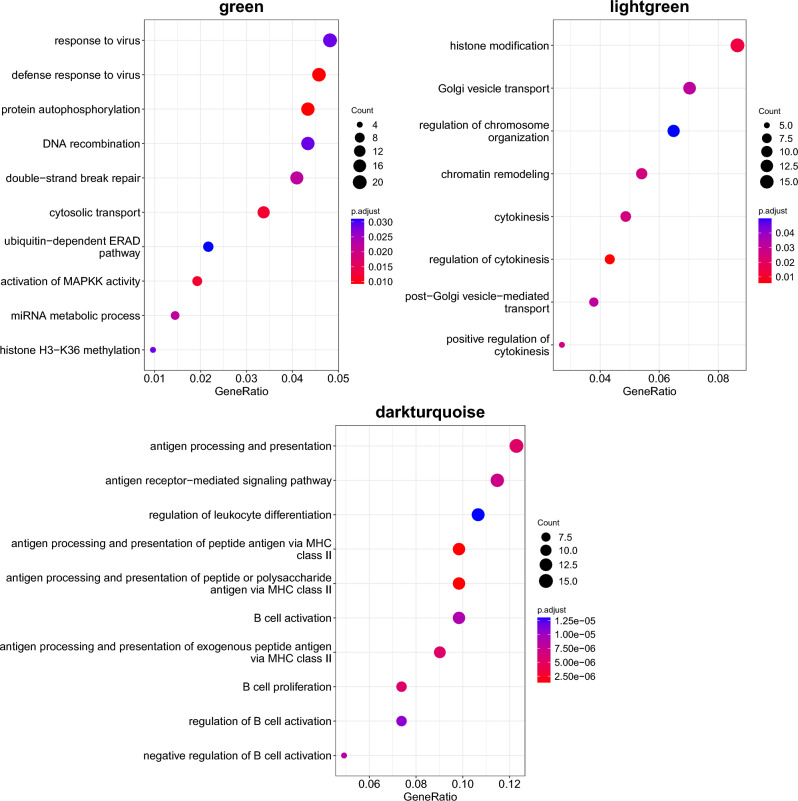
GO enrichment. Top 10 enriched Biological Processes in each asthma module. GeneRatio refers to the ratio between the genes in the module and the ones in the tested gene set.

**Figure 2:**
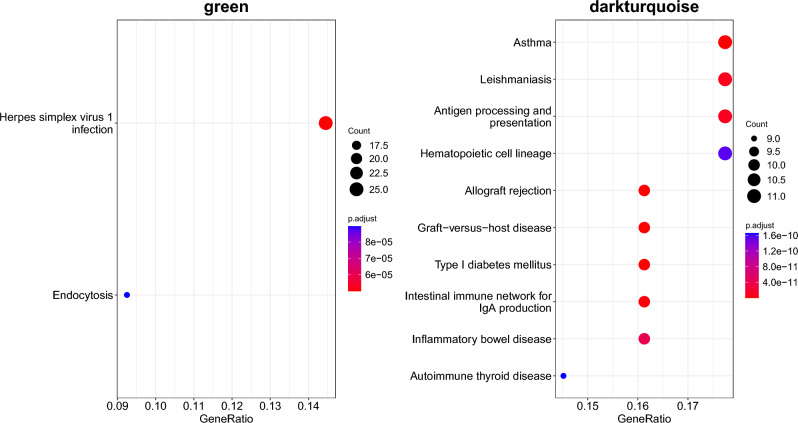
KEGG enrichment. Top 10 enriched KEGG pathways for each asthma module. The Lightgreen module did not show any enrichment.

Next, to identify the functional role of the asthma modules, we looked at the enrichment of genes in Biological Processes (BP) from Gene Ontology (Fig. 1) and KEGG pathways. The asthma modules were found to be enriched for BP related to response to viral infections (Green module), histone modifications and chromatin reorganisation (Lightgreen module) and antigen processing/presentation (Darkturquoise module) (Fig. 1 and Supplementary Data S1). The enrichment analysis for KEGG pathways identified enrichments for Herpes Simplex infection and endocytosis in the Green module and asthma and other pathways related to autoimmune diseases in the Darkturquoise module (Fig. 2 and Supplementary Data S1). No enrichment for KEGG pathways was identified in the Lightgreen module.

The Green module, a module enriched for pathways involved in the response to viral infections, was strongly enriched for childhood asthma and allergic disease traits GWAS-associated genes. The Darkturquoise module was enriched for antigen processing and presentation pathways, including the MHC class II genes *HLA-DRB5* and *HLA-DOB*, two genes previously associated with asthma^10,11^. The Lightgreen module was enriched for pathways involved in chromatin and histone modification, and positive regulation of cytokinesis. Interestingly, out of 10 top asthma-asociated genes in the Lightgreen module, 7 were either pseudogenes or long non-coding RNAs. Only one of these, *ENSG00000272477* (*Lnc-EFHB-6*) was reported in the literature as associated with the asthma-related trait eosinophil counts^12^, while no information could be found for the other 6. A result that highlights the importance of studying non-coding genes in asthma.

### Identification of master regulators of the asthma-associated modules

**Figure 3:**
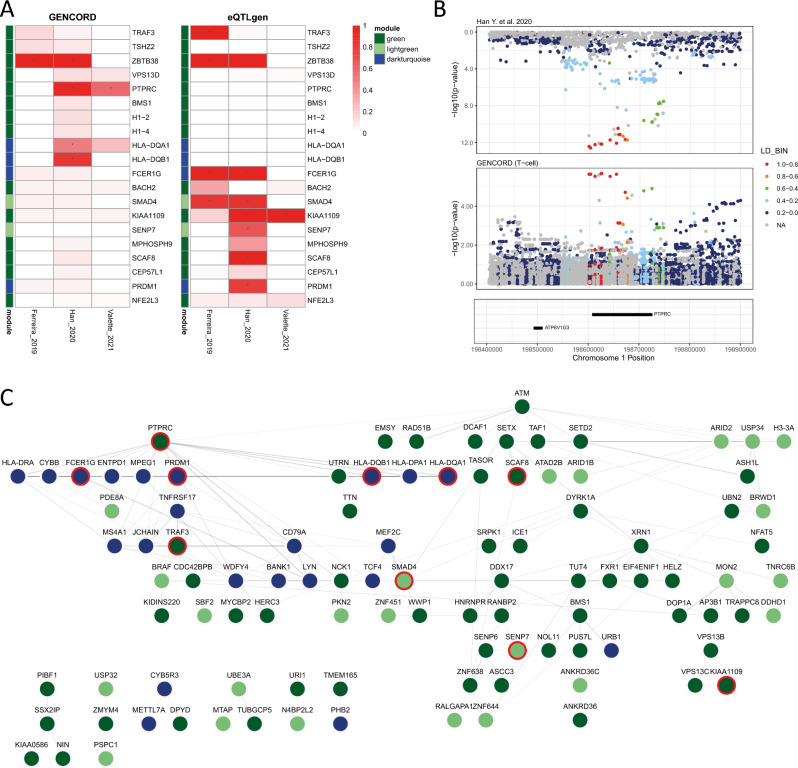
Identification of asthma-modules’ master regulators. (**A**) Result of the colocalisation analysis between asthma-GWAS and eQTL. For visualisation purposes, only results with PP4 ≥ 0.1 in any of the comparisons are shown. Asterisks represent a PP4 > 0.5; (**B**) mirrored locus zoom of the eQTL GENCORD and Han et al. 2020 asthma-GWAS signals for the PTPRC locus; (**C**) STRING’s protein–protein interaction network for the genes in the asthma-associated modules. Only genes with connections are shown. Nodes are coloured according to module names (Darkturquoise, Green and Lightgreen). Where present, the red outer-ring denotes evidence of GWAS-eQTL colocalisation.

It is difficult to disentangle the direction of effect between disease and differentially expressed genes, especially when taking into account the interaction between the pathways the genes belong to. Moreover, the WGCNA modules are comprised of hundreds of co-expressed genes, most of which would not have a genetically regulated component but, instead, regulated by the same genetically modulated master regulators, i.e. through eQTL. For this reason, we hypothesised that if the identified modules were a cause and not a consequence of the asthma pathogenesis, within the modules, we should have been able to identify genes whose expression has a genetically regulated component (i.e. co-localisation between eQTL and GWAS signals) and that are highly interconnected both within and between the disease-associated modules (hub genes). We therefore integrated publicly available eQTL and asthma-GWAS summary statistics. Colocalisation was used to identify signals with posterior probability of sharing a causal SNP (posterior probability 4 PP4) > 0.5 between eQTL and GWAS signals, restricted to genes in the asthma-associated modules (n = 886; see “Methods”, Supplementary Materials Fig. E9 and Supplementary Data S1 for full results). For the asthma-GWAS signal, three of the largest asthma-GWAS studies were used^6,10,13^. For the eQTLs, GENCORD^14^ (T-cell) and eQTLgen^15^ (whole blood) were used. GENCORD and eQTLgen identified different sets of genes with evidence of colocalisation with GWAS signals (Fig. 3A), with the majority of colocalised genes identified for eQTLgen (7 out of 11 unique genes). Only *ZBTB38* showed evidence of colocalisation in both datasets. The list of genes with evidence of colocalisation was then integrated with the top 10% quantile genes for module membership (MM—correlation between module eigengene and gene expression profile, a measure of association between the gene and the module) and gene significance (GS—correlation between gene expression and asthma, a measure of association between the gene and asthma) to include genes that are highly representative of the asthma-module expression profile (n = 165). This set of top genes was used as input in STRING to build a network with further evidence of protein–protein interaction. STRING identified *PTPRC* (a gene in the Green module with evidence of colocalisation, Fig. 3A,B) as the protein with the highest number of interactions and, therefore, as a putative master regulator of the identified asthma-associated modules (Fig. 3C; hub protein permutation p-value = 0.01, Supplementary Materials Fig. E10).

## Discussion

We presented the results of a Weighted Gene Co-expression Network Analysis on CD4^+^ T-cells in patients with well-controlled asthma and healthy controls. Three modules, named Green, Lightgreen and Darkturquoise, were associated with asthma status and were found to be strongly enriched for asthma and allergy related biological processes and KEGG pathways such as antigen processing and presentation, and immune response to viral infections. Importantly, the identified modules were also strongly enriched for asthma and childhood-asthma GWAS-identified genes, suggesting that the identified different expression profiles are partially genetically regulated by asthma-causal SNPs. In line with this observation, integration of publicly available asthma GWAS and blood eQTL summary statistics potentially identified a putative master regulator (*PTPRC*) of the asthma expression profiles with a genetically regulated component, possibly suggesting that the identified expression profiles are disease causing and not simply an asthma consequence.

WGCNA identified sets of co-expressed genes associated with asthma, and enriched for asthma and asthma-related traits, but only by identifying causal master regulators it is possible to further our understanding of asthma pathogenesis and to identify new druggable targets and asthma biomarkers. Moreover, from gene expression alone, it is not possible to infer the direction of effect between the studied phenotypes and the observed gene expression. The identified differences in gene expression might, in fact, be the consequence and not the cause of the studied phenotype. We, therefore, integrated mRNA co-expression (WGCNA), eQTL, GWAS and protein–protein interaction data to identify hub genes–genes highly interconnected within the network and therefore putative asthma master regulators—whose expression has a genetically regulated component, i.e. eQTL, by an asthma causal SNP, identified by GWAS studies. We identified *PTPRC*, a gene previously identified in GWAS studies, which also showed evidence of colocalisation with T-cell specific eQTL from GENCORD, as a putative master regulator in CD4^+^ T-cell asthma gene expression profile. Consistent with our findings (i.e. higher expression of *PTPRC* in asthmatics), eQTLs reported in GENCORD to be associated with increased expression of *PTPRC* are also reported to increase the risk of asthma in GWAS studies.

Similar to our study design, Do et al. used WGCNA to study differences in gene expression profiles from nasal brushings between healthy controls and severe asthmatics. They then applied a probabilistic causal network analysis to identify possible master regulators of the asthma-associated modules. Despite using a different analytical method, applied to a different cell type, they also identified *PTPRC* as a master regulator of a persistent asthma module, which was enriched in inflammatory response pathways^16^. Our study, therefore, builds on existing evidence that *PTPRC*, and its downstream gene pathway, is important in the asthma pathogenesis. It also suggests that the expression profiles observed in CD4^+^ T-cell from peripheral blood recapitulate those from tissue-resident CD4^+^. Valette et al., using a combination of functional mapping tools, also reported *PTPRC* as a candidate causal gene and a potential asthma drug target^13^.

*PTPRC* is a member of the protein tyrosine phosphatase family and encodes for the tyrosine phosphatase CD45. CD45, also known as leukocyte common antigen (LCA), plays an important role in T- and B-cell antigen receptor signal transduction via the Src family kinase Lck, and it is known to be associated with immunodeficiency^17^. CD45 has been shown to regulate several asthma related traits such as cytokine^18^, IL-6^19^ and IgE^20^ production but its role in the asthma pathogenesis has yet not been determined.

To date, only one other transcriptome study on asthma focused on CD4^+^ T cells. Tsitsiou et al. identified 40 differentially expressed genes (fold change > 1.5) between 12 severe asthmatics and 8 healthy controls^8^. While they also had data on 4 non-severe asthmatics, the small sample size did not allow them to properly study this subgroup. The observed small differences led the authors to conclude that severe asthma is not associated with the activation of circulating CD4^+^ T cells. Of the 40 DEGs genes identified by Tsitsiou et al. 5 were part of the asthma-associated modules such as *S100A9* (S100 Calcium Binding Protein A9), *S100A8* (S100 Calcium Binding Protein A8) and *MNDA* (Myeloid Cell Nuclear Differentiation Antigen) which belonged to the Darkturquoise module, i.e. lower expression in asthmatics. Consistent with our observation that *S100A9* and *S100A8* are under-expressed in CD4^+^ T-cells from asthmatics, knock-out mice for *S100A9* were shown to have increased Th2 cytokine levels and impaired Treg-mediated suppression of lung inflammation, compared to wild-type, after being challenged with the extracts from the allergenic mold *Alternaria alternata*^21^. *MNDA*, although not reported as associated with asthma, allergy or blood cell counts in GWAS studies, contains an interferon-stimulated response element in its 5’-UTR, and is reported to be an interferon induced gene^22,23^.

This study comes with several limitations. In line with Tsitsiou et al. findings^8^, the identified differences were generally small between the groups, which did not allow us to use conventional differential expression approaches. The fact that we did not identify any differentially expressed gene despite a larger sample size and a more sensitive technique than the one used by Tsitsiou et al. suggests that future asthma studies on CD4^+^ T cells should use even larger sample sizes or perform the analysis under stimulatory conditions. For this purpose, our study will serve as a reference for the required sample size. Nevertheless, our strict experimental design (using well-characterised asthma participants, who were followed up from birth until age 21 years and who showed asthma symptoms throughout their lives, the isolation of cells within 2 h from collection, the employment of a single operator from cell isolation to library preparation and the usage of UMIs) reduced non-biological heterogeneity to a minimum and, combined with sophisticated analysis techniques, such as WGCNA corrected for cell type proportions from bulk RNAseq deconvolution, allowed us to identify small differences between the groups.

A further limitation of the study is that asthma treatments, which could affect gene expression, were not taken into account in the analysis. Despite this, the strong enrichment for genes annotated to asthma, and asthma-associated traits, GWAS hits, and the identification of an asthma master regulator with a genetically regulated component suggest that the identified effects are at least partially genetically regulated and not a consequence of confounders.

In conclusion, CD4^+^ T-cells have been previously identified as a critically important cell type in asthma. Despite this, CD4^+^ T-cells have been greatly understudied. Using a network-based approach, we showed that, albeit small in magnitude, CD4^+^ T-cells from participants with well-controlled asthma exhibit a different expression profile compared to healthy controls and that the three asthma-associated modules are strongly enriched for genes identified through GWAS studies. By integrating publicly available genetic and protein–protein interaction data, we identified *PTPRC*, a gene previously identified as a master regulator of asthma gene expression profile from nasal brushing, as a master regulator in peripheral CD4^+^ T-cells. The identification of a CD4^+^ T-cell master regulator provides an important step towards the discovery of new druggable targets and possible biomarkers for asthma diagnosis. Finally, our results can also be used as a reference for future, better powered studies aiming at identifying differences between people with well-controlled asthma and healthy controls.

## Methods

A more detailed description of the methods can be found in the Supplementary Materials S1.

### Participants

Participants from the MAAS cohort^9^, who showed asthma symptoms throughout their lives and who have had asthma symptoms within 12 months prior to the year 18 + visit were recruited into the asthma cases group (n = 33), while participants with no prior history of asthma were recruited as control subjects (n = 12). The study was approved by the North West—Greater Manchester East Research Ethics Committee and it was performed in accordance with relevant guidelines and regulations. Informed consent was obtained from all participants and/or their legal guardians.

### Cell isolation and RNA extraction

CD4^+^ T-cells were isolated from blood by negative selection within 2 h from collection. The RNA was extracted using the RNeasy mini extraction kit and processed according to the manufacturer’s protocol (Qiagen). All the samples had RNA integrity number (RIN) score > 9 and were taken forward for downstream processing.

### Library preparation and sequencing

Libraries were generated with the QuantSeq 3' mRNA-Seq Library Prep Kit (Lexogen) and the protocol was followed without modifications. Samples were split into 4 batches for library preparation, balanced by sex and case–control status, pooled together and sequenced on a single NextSeq4000 flow cell (single-end 150 bp) to an average read depth of ~ 8 million reads.

### Read mapping and UMI deduplication

Reads were quality trimmed with Trimmomatic^24^ and poly-G and poly-A tails were removed with Cutadapt^25^. FastQC was used to assess read quality, before and after trimming, and duplication rate. STAR^26^ was used to map the reads (hg38) using the parameters as recommended by Lexogen bioinformatics support. Reads mapping to the same genomic coordinates were deduplicated based on UMI sequence with UMI-tools^27^ and gene counts were calculated with HTSeq^28^.

### QC and weighted gene co-expression network analysis (WGCNA)

Only genes with at least 5 counts in at least 10 samples were retained for further analysis. Hierarchical clustering of samples using the top 100 most expressed genes was used to identify outliers. Post-hoc power calculation was carried out with the R package RnaSeqSampleSize. Read counts were normalised by sequencing depth and transformed with VST using DESeq2 v3.14^29^. DESeq2 was used for differential expression analysis. A weighted gene co-expression network was built using the top 75% most variable genes with WGCNA^30^ (see Supplementary Materials S1). Sex, sequencing batch, number of PCR cycles and cell type proportions (for cell with average proportions > 5%) were included as covariates in the DESeq2 model and were regressed out prior to WGCNA (Supplementary Materials Figs. E3, E4). A network of type “signed hybrid” was chosen (soft threshold = 6), i.e. only positive correlations between the genes were used to build the network. Sets of highly co-expressed genes were then grouped into modules. The average expression profile of each module was calculated (moduleEigengenes).

The per-module eigengene values were used to test for differences between asthmatics and non-asthmatics using logistic regression. Given the small differences in gene expression observed from the DESeq2 analysis, an FDR of 20% was considered significant and results were further investigated.

### Bulk RNA-seq decomposition

To correct for differences in the abundance of specific CD4^+^ sub-populations between samples and/or for CD4^+^ isolation efficiency, the cell-type abundance of different CD4^+^ T cells was estimated using the BisqueRNA^31^ R package. Single-cell RNA-seq gene counts were extracted from three T-cell datasets (SRA814476, SRA794656, SRA665712) using the R package rPanglaoDB^32^. Seurat^33^ was used to label the cells by mapping the datasets to the Seurat-provided annotated CITE-seq references. The R package BisqueRNA^31^ was used to estimate cell type proportions. The cell type proportions of cells with average proportions > 5% across samples were used as covariate for WGCNA module association with asthma (see Supplementary Materials Figs. E7, E8).

### Over representation analysis (ORA)

Modules were tested for enrichment (Fisher’s exact test) in Gene Ontology (GO) Biological Processes (BP) and KEGG^34^ pathways using ClusterProfiler^35,36^. Enrichment for GWAS Catalog^37^ traits was assessed using FUMA^38^.

### Colocalisation of eQTL and asthma GWAS signals for the asthma-associated modules’ genes

Summary statistics of three of the largest asthma GWAS studies were downloaded^6,10,13^. The lead-SNP for each study was obtained from the GWAS catalog. The GWAS hits (and their proxy SNPs; R^2^ ≥ 0.7 in the European population 1000G phase III) were annotated to protein-coding genes based on the following criteria: (1) the SNP position overlapped the gene body coordinates; (2) closest preceding/following gene; (3) the SNP position overlapped a locus that interacted with the gene promoter in CD4^+^ T-cells (promoter capture Hi-C interaction matrix from Javierre et al.^39^). The annotated GWAS hits were then filtered to only retain signal for genes present in the asthma-associated modules. The eQTL summary statistics for GENCORD^14^ and eQTLgen^15^ were obtained from the European Bioinformatics Institute and eQTLgen websites, respectively. GWAS and eQTL summary statistics were filtered to retain only signals involving genes from the asthma-associated modules. For the regions with overlapping signals between the GWAS and eQTL datasets, the R package Coloc^40^ was used to test for colocalisation (Supplementary Materials Fig. E9).

### Identification of master regulators within the asthma-associated modules

To identify the asthma-gene master regulators, a set of top asthma-genes was constructed: (1) genes with a posterior probability (PP4) of a shared causal SNP between eQTL and GWAS summary statistics ≥ 0.5; i.e. genes whose expression has a genetically regulated component by an asthma causal variant; (2) top 10% quantile of genes based on module membership (MM—correlation between module eigengene and gene expression profile; i.e. genes strongly representative for the module expression profile); (3) top 10% quantile of genes based on gene significance (GS—correlation between gene expression and asthma; i.e. genes strongly associated with the asthma phenotype). A protein–protein interaction network (PPI) was built using this list of genes with STRING^41^ and visualised in Cytoscape v3.9.1^42^. Since the set of genes was derived from WGCNA modules, to avoid redundancy of information, the STRING interaction sources “co-expression”, “neighbourhood” and “gene fusion” were deselected. The protein with the largest number of PPI (number of edges in the network—hub protein) and with evidence of colocalisation was considered a putative master regulator. To determine the significance of the identified hub protein, we compared the number of edges of the identified hub protein to the distribution of number of edges of “hub proteins” from random gene sets. Starting from the combined set of proteins from the asthma-associated modules, we build 10 thousand PPI networks from random sets of the same size as the set of top asthma-genes. The distribution of the maximum number of edges from each permutation was then used to estimate the hub protein p-value.

### Supplementary Information

Supplementary Information 1.

Supplementary Information 2.

Supplementary Information 3.

## Data Availability

The datasets generated and/or analysed in the current study have been deposited in NCBI's Gene Expression Omnibus (GEO) and are accessible through GEO Series accession number GSE217904.

## Abbreviations

GWAS: Genome-wide association study
SNP: Single nucleotide polymorphism
WGCNA: Weighted gene co-expression network analysis
GS: Gene-significance (i.e. gene-phenotype correlation)
GM: Gene-membership (i.e. gene-module correlation)
PTPRC: Protein tyrosine phosphatase receptor type C
MAAS: Manchester asthma and allergy study
eQTL: Expression quantitative trait loci
