# Supplementary material for: Identification of differences in CD4+ T-cell gene expression between people with asthma and healthy controls
Source: Sci Rep. 2023 Dec 20;13:22796. doi: 10.1038/s41598-023-49135-9 (PMC10739740; doi:10.1038/s41598-023-49135-9)
Supplement: Supplementary file 3 — Supplementary Information 3. [file 41598_2023_49135_MOESM3_ESM.docx]

**Study population**

The Manchester Asthma and Allergy Study (MAAS)

MAAS (www.maas.org.uk) is a population based birth cohort study designed to investigate the genetic and environmental factors involved in the development of asthma and allergies in childhood (Custovic, Simpson, and Woodcock 2004). More than 1000 participants were recruited prenatally between 1995 and 1997 by approaching the parents at the antenatal visits. Children attended follow-up clinics every 2 or 3 years from the age of 1 to 18 years (1, 3, 5, 8, 11, 13-16, 18+) for assessments, which included lung function measurements, skin prick testing, biological samples (serum, plasma and urine), and questionnaire data collection. DNA was extracted from whole blood where available or from Oragene samples. The study was approved by the North West – Greater Manchester East Research Ethics Committee.

**CD4^+^ T-cell isolation and library preparation**

Cell Isolation

Blood from recruited participants was collected in 4x10ml EDTA tubes and processed within 2 hours from collection for PBMC extraction. PBMC were isolated using 50mL Leucosep tubes and Ficoll-paque. PBMC pellets, at 50M cells per mL concentration, were resuspended in PBS (2% FBS, 2mM EDTA). CD4+ Enrichment Kit Easysep (Stemcell) was used to isolate CD4+ T-cells. A minimum of 1.5 x 10^6^ cells were transferred to a fresh Eppendorf and spun at 400g and 4°C for 5 minutes. The supernatant was removed and the cells were lysed by adding 700μL of QIAzol. The cells were stored at -80°C until ready for RNA isolation.

RNA extraction

700μL of QIAzol cell lysate was mixed with 140μL chloroform in a 1.5mL centrifuge tube (Starlabs), shaken well, and incubated at RT for 2-3 minutes. The samples were then centrifuged for 15 minutes at 12000xg at 4°C. The upper aqueous layer was transferred to a clean 1.5mL tube and mixed with 525μL 70% ethanol. The sample was then transferred into an RNeasy mini RNA extraction kit spin column (Qiagen) and processed according to the manufacturer’s protocol. The optional DNase treatment step detailed in the kit protocol was included. Quality of the RNA was assessed using the Agilent Tapestation 4150. All the samples had a RIN score of > 9 and were taken forward for downstream processing.

Lexogen system library preparation and sequencing

Samples were split into 4 batches balanced by sex and case-control status. QuantSeq 3' mRNA-Seq Library Prep Kit FWD for Illumina (Lexogen) was followed without any modifications. Briefly: between 100ng and 250ng of RNA was used for each subject. RNA was denatured and then reverse transcribed using oligo-dT primer containing Illumina Read2 linker sequence. The RNA strand was removed and the second cDNA strand synthesis was performed using random primers containing Illumina Read 1 linker sequence and the 6 nucleotide Unique Molecular Identifiers (UMI). The sample was then purified using magnetic beads supplied by the manufacturer. The recommended qPCR was performed to best estimate the number of PCR cycles required for the final amplification step where the indexes were incorporated. The entire process was done in a sterile RNase free environment.

Before sequencing, the quality of the libraries was assessed with Agilent Bioanalyser and the library concentration was quantified with Qubit. Libraries were then pooled to a final concentration of 12nM and sequenced on a single NextSeq4000 flow cell using single-end 150bp reads.

**Results**

QC

The percentage of unique and duplicated reads was estimate using FastQC. One sample with duplication > 90% was excluded from subsequent analyses.

Hierarchical clustering on the top 100 most expressed genes identified one sample as a possible outlier (Figure E1). Following a review of the lab notes the sample was identified as the only one not processed by the same operator.

Figure E1: hierarchical clustering of samples using the top 100 most expressed genes

Heatmap and hierarchical clustering of the samples (over columns) using the top 100 most expressed genes (over rows). Each cell represents the gene read counts normalised by sequencing depth and transformed with the DESeq2 vst function. Red arrow highlights the sample outlier. Heatmap created with DESeq2 v 3.14.


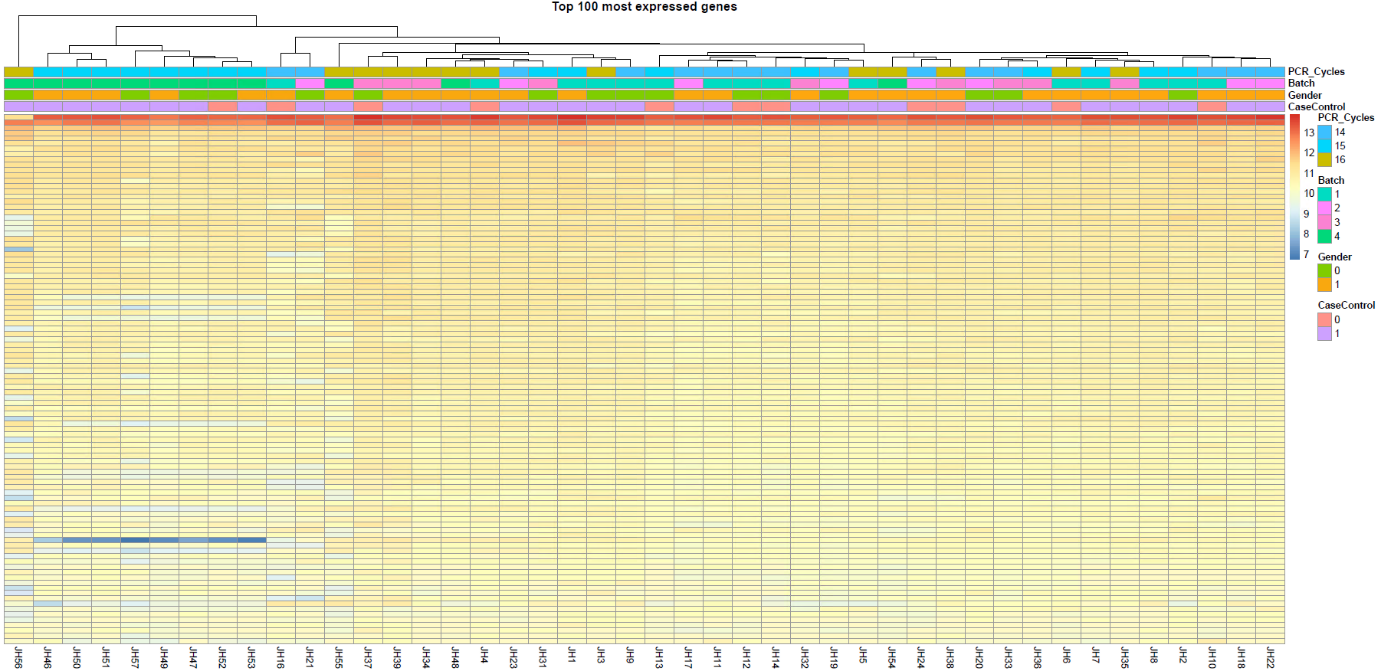


Differential expression

DESeq2 was used to test for differential expression between asthmatics and healthy controls while controlling for sex, number of PCR cycles used during library preparation, library batch number and cell type proportions (for cell with average proportions > 5%, see “Bulk RNA-seq decomposition” section). Only genes with at least 5 reads in at least 10 samples were used in the analysis. No significant differentially expressed gene was identified (Figure E2).

Figure E2: Volcano plot of DESeq2 results for asthmatics vs healthy controls. Horizontal line denotes p-value=10e-6 (FDR=0.05); vertical lines denote log2FC=1.5

Post-hoc power calculation

Given the very small fold changes observed between asthmatic patients and healthy controls, we performed a post-hoc power calculation using the R package RnaSeqSampleSize. The gene count dispersion for the current dataset was calculated using the function est_count_dispersion. The functions est_power_distribution(n=43, f=0.05, rho=1.5) and sample_size_distribution(power=0.8, f=0.05, rho=1.5, k=0.34) were used to estimate the power for the current analysis and the sample size that would have been needed to obtain 80% power detect differentially expressed genes with fold change = 1.5. The analysis revealed that we only had 69% power and that a sample size of at least n=52 would have been needed.

Weighted Gene Co-expression Network Analysis (WGCNA)

To build the weighted gene co-expression network, genes in the bottom quartile based on the variance (e.g. non-variable genes) were removed from the analysis. The read count matrix was then normalised with DESeq2 VST function. Batch effects from sex, sequencing batch and number of PCR cycles were removed using the function removeBatchEffect from the R package limma. The R package WGCNA (v1.7) was then used to build the network. To build a network of co-expressed genes, a pair-wise distance matrix was first calculated as a measure of co-expression. For this step a robust measure of correlation, the biweight mid-correlation, which uses the median instead of the mean and it is therefore more robust to outliers, was used. The distance, or similarity, matrix was then transformed into an adjacency matrix by raising the matrix to a power n. The aim of this transformation is to obtain a network that resembles as much as possible a scale-free network. To determine the best n, the function pickSoftThreshold was used to calculate adjacency matrices from a set of powers ranging from 1 to 20. The network type was set to “signed hybrid” which converts negative correlations to zero. The scale-free topology fit (R^2^ value; Figure E3) and the mean connectivity values (Figure E4) were plotted as a function of the soft-threshold powers to determine the best fit. A power of 6 was chosen to build the final network being the one with the highest R^2^ and lowest mean connectivity value (inflexion point). Starting from the adjacency matrix, a Topological Overlap Matrix (TOM) was constructed using the function TOMsimilarity and converted into a dissimilarity matrix (1 – TOM). The tree branches of the hierarchical clustering, built from the dissimilarity matrix, were dynamically cut using the function cutreeDynamic to identify clusters of co-expressed genes using the parameters deepSplit = 2, pamRespectsDendro = FALSE and minClusterSize = 30. Finally, the gene expression profile of each cluster was summarised by the first principal component, also called eigengene, using the function moduleEigengenes. Highly correlated clusters were merged together into a final set of clusters, also called modules, by building a hierarchical clustering tree on the dissimilarity measure calculated from the clusters’ eigengenes and merging those that were more than 75% similar.


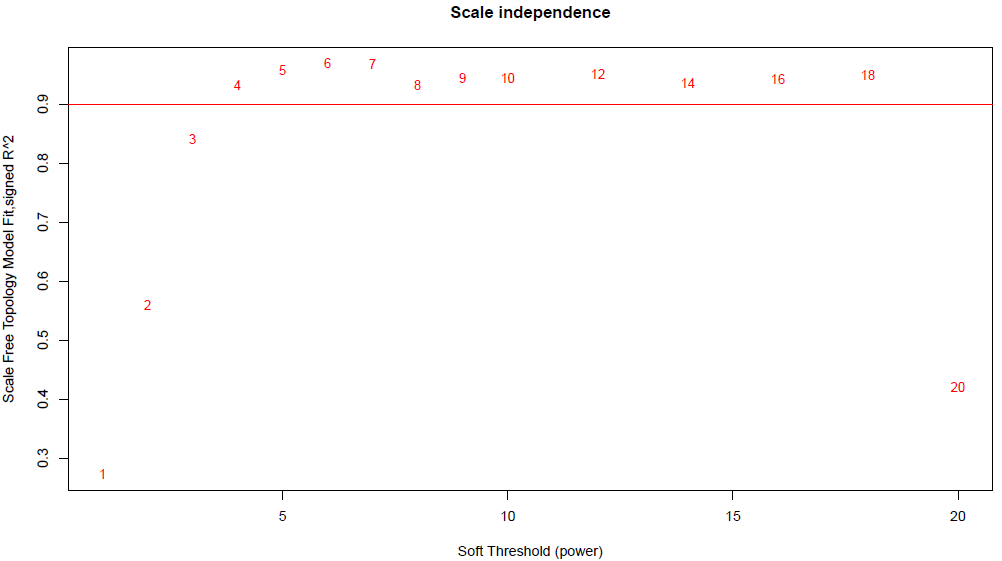


Figure E3: Scale-free topology fit index as a function of the soft-thresholding power

The plot shows how well the network model built with soft thresholding power ranging between 1 and 20 (x-axis) fit a scale-free model (R^2; y-axis)


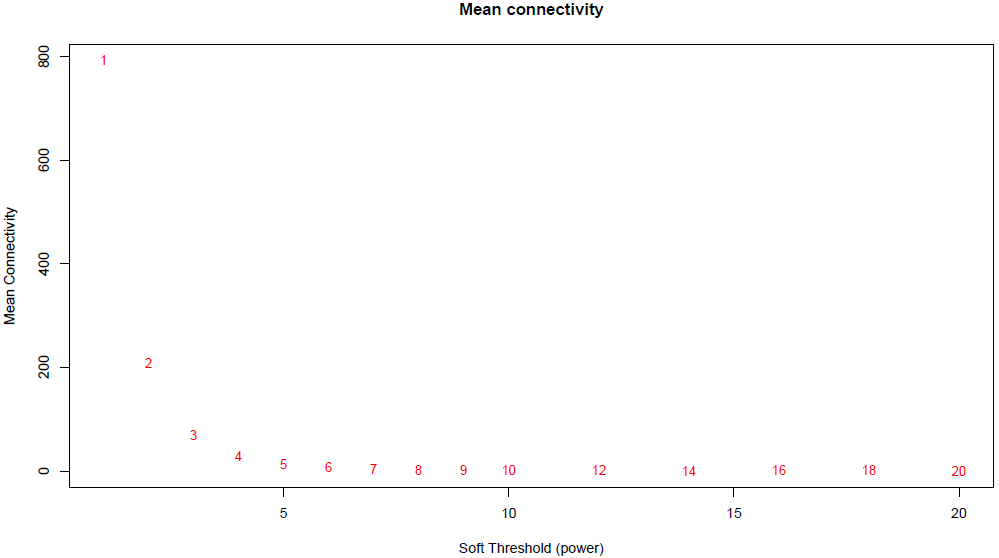


Figure E4: Mean connectivity as a function of the soft-thresholding power

The plot shows that mean number of the network’s nodes connections as a function of the chosen soft thresholding power

WGCNA identified 18 modules of co-expressed genes plus one module of genes not belonging to any other module (singletons). Each module was assigned a colour code with the colour grey reserved for singletons and not used in the following analyses. The size of the modules ranged from 104 to 1450 genes (Table E1).

| **Module** | **N. genes** |
| --- | --- |
| turquoise | 1450 |
| blue | 1212 |
| black | 974 |
| grey60 | 940 |
| brown | 754 |
| midnightblue | 648 |
| greenyellow | 610 |
| green | 490 |
| magenta | 332 |
| purple | 312 |
| tan | 308 |
| lightcyan | 270 |
| lightgreen | 252 |
| royalblue | 212 |
| darkred | 164 |
| darkgreen | 159 |
| darkturquoise | 144 |
| darkgrey | 104 |

Table E 1: WGCNA module name and number of genes in each module

Figure E5: **Graphical representation of the relationship between the WGCNA modules**

A) Dendrogram of the GWCNA modules based on dissimilarity between the modules eigengene values. B) heatmap of the eigengene adjacency (similarity measure) between modules. Heatmap created with WGCNA R package v1.70.


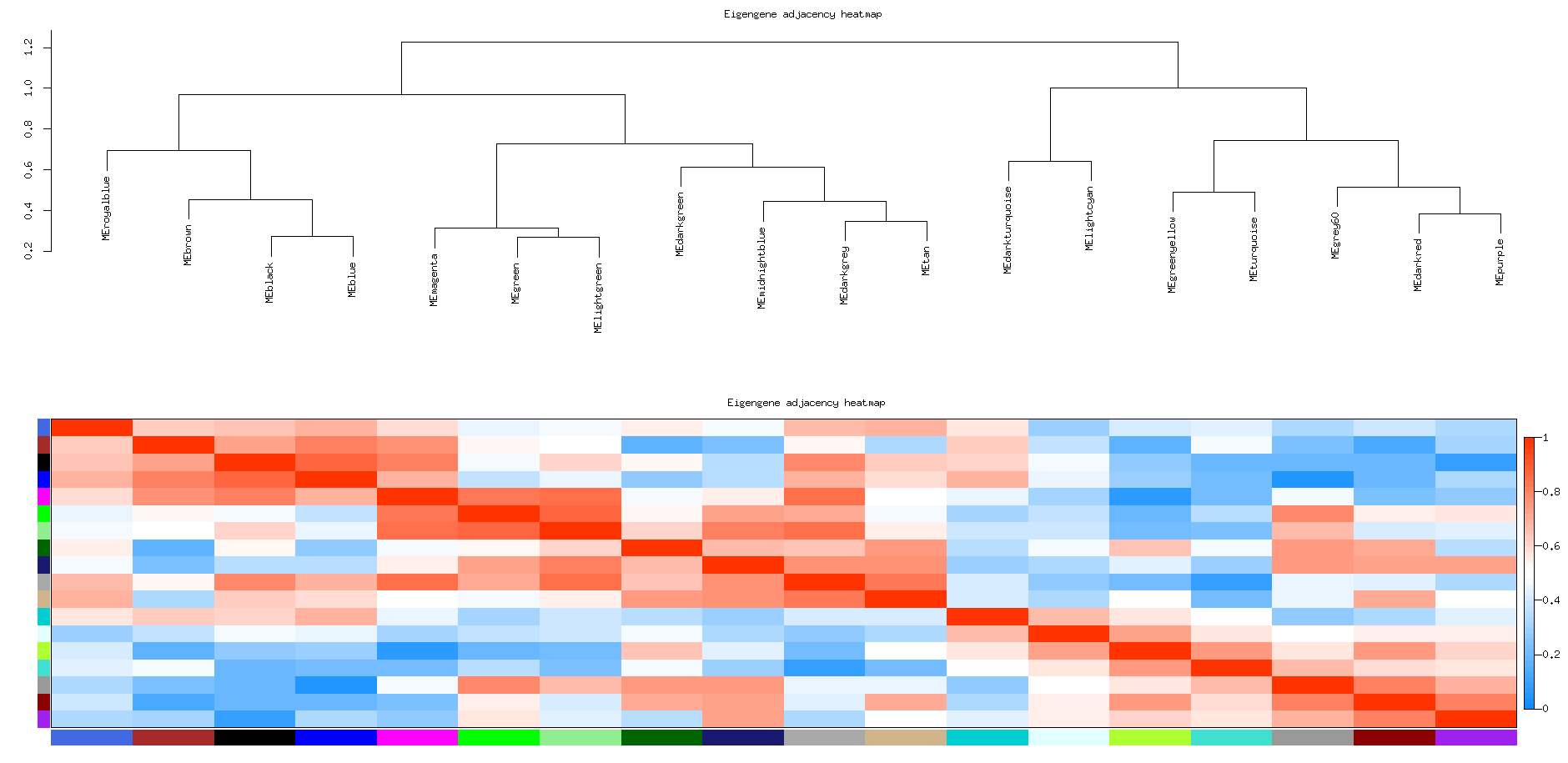


A

B

Figure E6: **Boxplot of normalised gene counts of the top 10 hub genes in the asthma associated WGCNA modules**. Genes with unknown gene symbol were referred to with their Ensembl gene ID


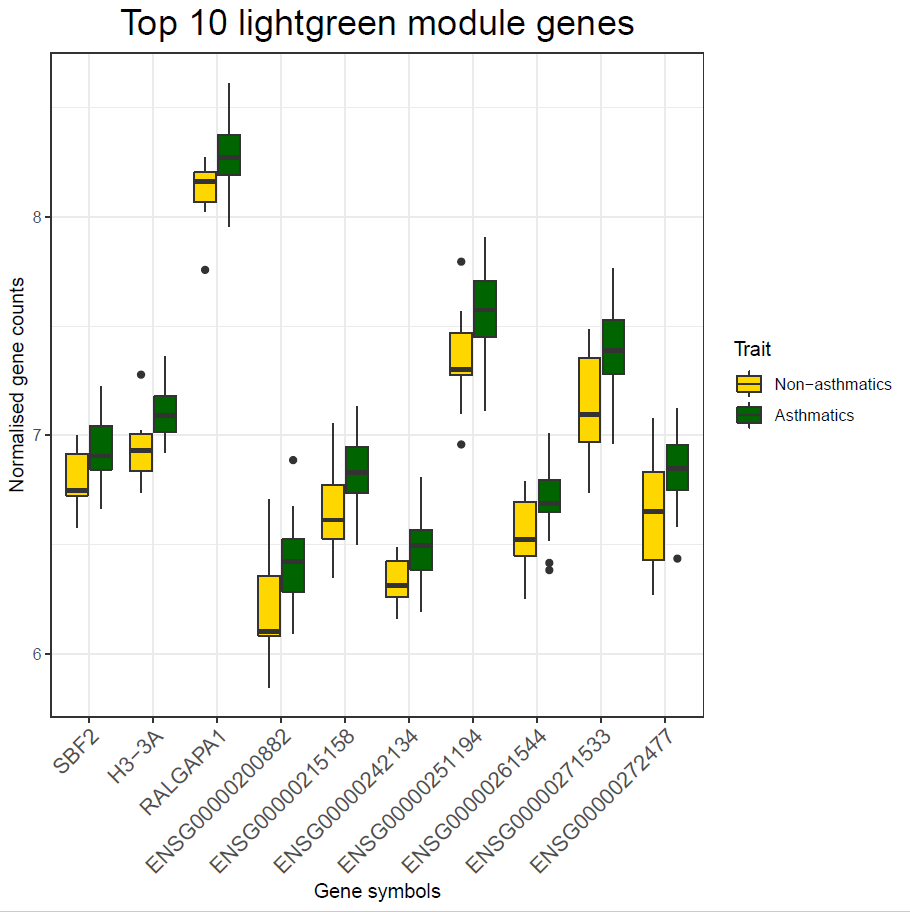

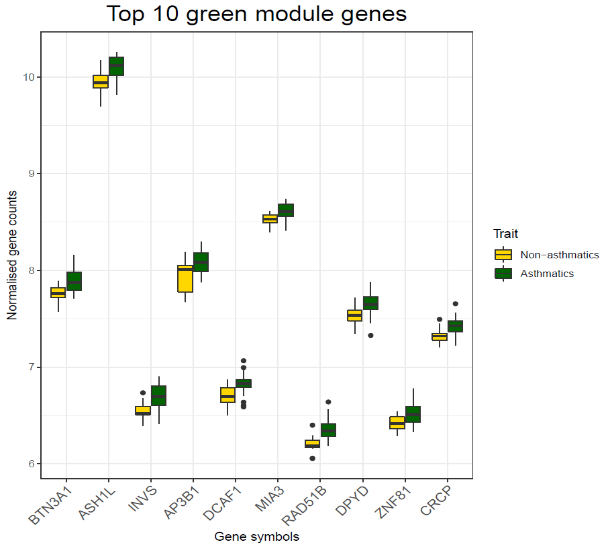

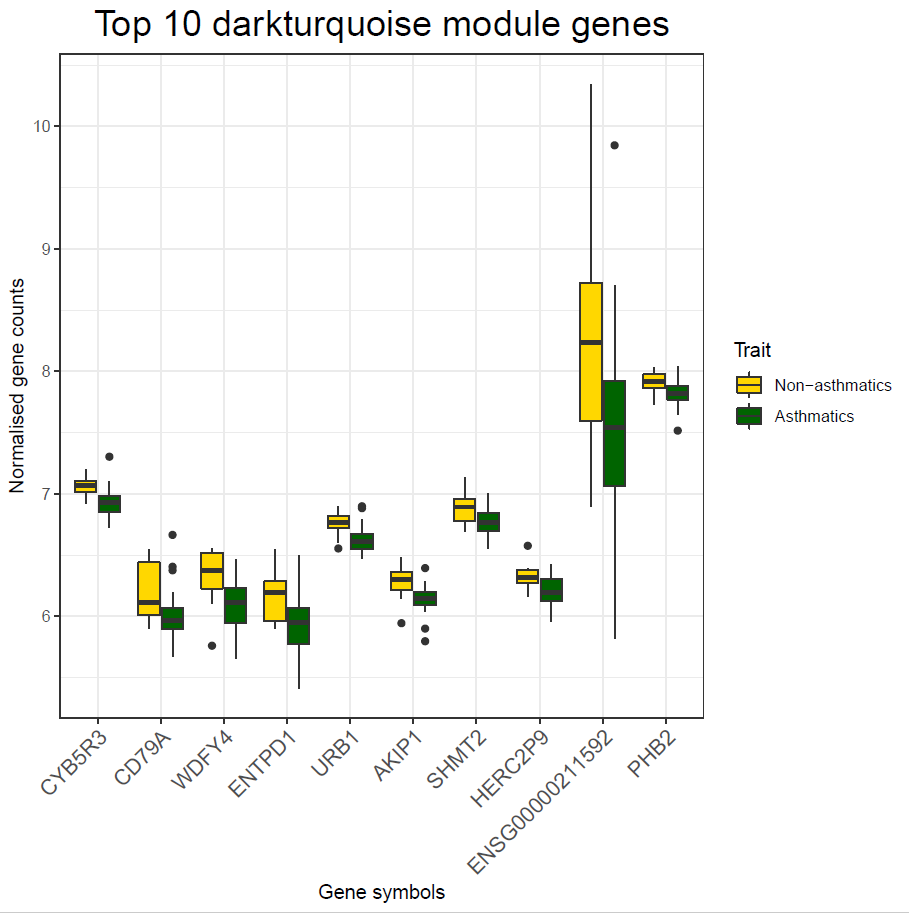


| Module | Gene | Study | SNP | p-value | Trait |
| --- | --- | --- | --- | --- | --- |
| green | *BTN3A1* | UKBB | rs41266839 | 3x10-08 | Asthma |
|  | *ASH1L* | UKBB | rs61812092 | 2x10-08 | Hayfever, allergic rhinitis or eczema |
|  | *INVS* | UKBB | rs576094032 | 8x10-06 | Status asthmaticus |
|  | *AP3B1* | GCST007429 | rs376416 | 6.3x10-14 | Lung function (FVC) |
|  | *DCAF1(VPRBP)* | UKBB | rs73078636 | 7.x10-08 | Asthma |
|  | *MIA3* | UKBB | rs188380685 | 1x10-06 | Status asthmaticus |
|  | *RAD51B* | GCST009720 | rs1950897 | 8x 10-13 | Asthma |
|  | *DPYD* | GCST009417 | rs6703307 | 2x 10-6 | HIV progression (CD4 and viral load) |
|  | *ZNF81* | none | none | none | none |
|  | *CRCP* | GCST90002389 | rs1404147 | 3.8x10-9 | Lymphocyte percentage of white cells |
| lightgreen | *SBF2* | GCST007080 | rs78399419 | 3x 10-8 | Lung function (FEV1/FVC) |
|  | *H3-3A* | none | none | none | none |
|  | *RALGAPA1* | none | none | none | none |
|  | *ENSG00000200882* | none | none | none | none |
|  | *ENSG00000215158* | none | none | none | none |
|  | *ENSG0000024134* | none | none | none | none |
|  | *ENSG00000251194* | none | none | none | none |
|  | *ENSG00000261544* | none | none | none | none |
|  | *ENSG00000271533* | none | none | none | none |
|  | *ENSG00000272477* | GCST90002381 | rs6550653 | 4x 10-10 | Eosinophil counts |
| darkturquoise | *CYB5R3* | none | none | none | none |
|  | *CD79A* | none | none | none | none |
|  | *WDFY4* | UKBB | rs188070643 | 1x10-09 | Treatment with zirtek allergy 10mg tablet |
|  | *ENTPD1* | GCST90001484 | rs2861152 | 1x10-311 | Lymphocyte count |
|  | *URB1* | UKBB | rs184468695 | 3x10-08 | Treatment with zirtek allergy 10mg tablet |
|  | *AKIP1* | none | none | none | none |
|  | *SHMT2* | UKBB | rs74093117 | 1x10-06 | Asthma |
|  | *HERC2P9* | none | none | none | none |
|  | *ENSG00000211592* | none | none | none | none |
|  | *PHB2* | none | none | none | none |

Table E 2: **Reported associations with asthma associated hub genes in the GWAS Catalog and UK Biobank (UKBB) databases**. Only association with asthma, allergy and infection related traits at p-value < 10-5 are reported. For GWAS Catalog association we report the study accession number. None=no reported associations

**Bulk RNA-seq decomposition**

Cell-type abundance of different CD4^+^ T cells was estimated using the BisqueRNA R package. The R package rPanglaoDB was used to extract single-cell RNA sequencing (scRNA-seq) gene counts from 3 T cell datasets (SRA814476, SRA794656, SRA665712). The R package Seurat was then used to label the cells by reference mapping. Finally, this reference scRNA-seq was used to estimate the cell type abundance of the bulk RNA-seq. As expected, CD4^+^ T cell were estimated to be the most abundance cell population (Figure E7, E8). Logistic regression showed no evidence of association between asthma and cell type proportions (data not shown).

Figure E7: **Cell type proportion by condition – low granularity**

Figure E8: **Cell type proportion by condition – high granularity**

Modules that were previously identified as associated with asthma were tested again including the cell type abundance (only for cell types with estimated abundance > 5% - CD4 Naïve, CD4 TCM, gdT and Treg) as a covariate in the logistic regression model formula:

$$logit\left( \mathrm{Condition} \right)=\beta0+ moduleEigengene*\beta1 + CellTypeAbundance_{1}*\beta2+ \mathrm{CellTypeAbundanc}e_{2}*\beta3+ \mathrm{CellTypeAbundanc}e_{n}*\beta_{n}$$

Sensitivity analyses

**House dust mite skin prick test (HDM SPT)**

To test whether the identified association were specific for asthma or driven by atopy, the results from HDM SPT at age 18 years were included as a covariate in the logistic regression models. Controlling for atopy in the model did not change the estimates (Table E6) and, therefore, the results are likely asthma specific.

|  | No covariates | | With HDM SPT covariate | |
| --- | --- | --- | --- | --- |
|  | beta | p-value | beta | p-value |
| Green | 4.96 | 0.043 | 5.64 | 0.037 |
| Lightgreen | 4.94 | 0.045 | 4.70 | 0.065 |
| Darkturquoise | -4.08 | 0.084 | -4.94 | 0.058 |

Table E3: **Sensitivity analysis of modules association with asthma – house durst mite skin prick test [HDM SPT] age 18 years**. Results of associations of modules expression profiles with asthma with or without the inclusion of the HDM SPT results at age 18 years in the logistic models. The covariates sex and number of PCR cycles were included in both analyses.

Colocalization of eQTL and asthma GWAS signals for the asthma-associated modules’ genes

GWAS summary statistics:

- Ferreira et al 2019
- Han et al 2020
- Valette et al 2021

Lead SNPs (pval 5e-8) expansion:

- Proxy SNPs (LD R^2^ > 0.7)

SNPs mapped to genes:

- SNP overlapping gene coordinates
- Closest gene preceding SNPs
- Closest gene following SNPs
- Gene promoter interacting with SNPs (Javierre et al 2016 PCHiC) – CD4+ only

Filter for genes in the WGCNA asthma-associated modules

**Colocalization of signal**

eQTLgen summary statistics:

- eQTLgen (whole blood)
- GENCORD (T-cell)

Figure E9: **Schematic representation of asthma GWAS and eQTL data with WGCNA asthma-associated modules**

PTPRC

Figure E10: **Number of edges distribution from permutation**. Histogram of the distribution of the number of edges of the “hub protein” from each of 10K permutations obtained from STRING. The dashed line represents the number of edges for PTPRC.
